# Mass Cytometry‐Based Approach for the Investigation of Stimulator of Interferon Genes Pathway

**DOI:** 10.1002/eji.70101

**Published:** 2025-12-18

**Authors:** Anne Reversat, Paul T. Kennedy, Anni Georghiou, Joseph R. Slupsky, Lekh N. Dahal

**Affiliations:** ^1^ Department of Pharmacology and Therapeutics University of Liverpool Liverpool UK; ^2^ Department of Molecular and Clinical Cancer Medicine University of Liverpool Liverpool UK

**Keywords:** immunotherapy, innate immunity, mass cytometry, STING

## Abstract

The stimulator of interferon genes (STING) pathway plays a pivotal role in innate immunity, acting as a key sensor of cytosolic DNA to initiate type‐I Interferon (IFN) and pro‐inflammatory cytokine production. This pathway is essential for host defence against bacterial, viral and other pathogenic threats and has emerged as a promising therapeutic target in cancer immunotherapy. However, conventional techniques such as immunoblotting and qPCR are limited in their capacity to study STING pathway activation in complex and heterogeneous biological systems, such as tumour masses or large cell populations. Here, we describe the application of mass cytometry (CyTOF) as a cutting‐edge approach to characterize the STING pathway at the sub‐population level. Using a high‐dimensional panel of metal‐labelled antibodies targeting key STING signalling components, we achieved resolution of pathway activation across diverse immune cell populations. This approach promises novel insights into cellular heterogeneity, pathway dynamics and the interplay between STING signalling and other immune pathways and underscores the power of high‐dimensional analysis to overcome the limitations of traditional methods to enable a more comprehensive exploration of immune signalling pathways.

## Graphical abstract

Using mass cytometry, we map the heterogeneous activation of the STING pathway at single‐cell resolution in cell lines and primary human immune cells and validate them with conventional techniques.

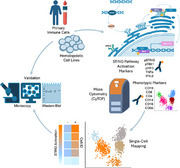

## 1 Introduction

The stimulator of interferon genes (STING) pathway is a critical component of the innate immune system, serving as a central mechanism in the host's defence against bacterial, viral and other pathogenic threats [1, 2]. Activation of the STING pathway occurs in response to cytosolic DNA and cyclic dinucleotides, triggering a cascade of signalling events that culminate in the production of type‐I interferons (IFNs) and pro‐inflammatory cytokines [1, 2]. This immune response plays a pivotal role in pathogen clearance and the maintenance of cellular homeostasis [3]. Beyond its role in infection, the STING pathway has emerged as a promising therapeutic target in the field of oncology [4, 5]. Its ability to stimulate anti‐tumour immunity has spurred the development of STING agonists and modulators as potential cancer immunotherapies [6, 7, 8]. However, while the biological significance of the STING pathway is well‐recognized, current methodologies for its characterisation remain constrained by technical limitations.

Conventional techniques such as immunoblotting and quantitative PCR (qPCR) have been widely employed to investigate the STING pathway. These methods, while robust and informative, are inherently limited in their ability to provide a comprehensive analysis of the pathway within complex biological systems. For instance, immunoblotting offers insights into protein expression and post‐translational modifications, but it is low throughput. Similarly, qPCR enables the quantification of gene expression but lacks the resolution to discern pathway dynamics at the single‐cell level. These traditional approaches are particularly inadequate for studying the STING pathway within heterogeneous cell populations, such as those found in tumour microenvironment or during immune responses to infections. The inability to capture the functional heterogeneity and intricate cellular interactions within large cell populations underscores the need for innovative methodologies that can overcome these limitations.

Mass cytometry, also known as cytometry by time‐of‐flight (CyTOF), represents a transformative advancement in the field of high‐dimensional single‐cell analysis. By integrating principles of flow cytometry with mass spectrometry, mass cytometry enables the simultaneous measurement of over 40 parameters at the single‐cell level [9]. This unparalleled capability facilitates an in‐depth examination of cellular phenotypes, signalling pathways and functional states within complex biological systems [10]. Unlike conventional techniques, mass cytometry can resolve cellular heterogeneity and identify distinct subpopulations within a sample, thereby providing a more nuanced understanding of STING pathway activation and regulation.

In this study, we harnessed the power of mass cytometry to characterize the STING pathway within diverse cell lines and immune cell populations. By leveraging a panel of metal‐tagged antibodies targeting key proteins involved in the STING signalling cascade, we achieved high‐dimensional profiling of pathway activation across multiple cell types and validated it with conventional techniques. This approach enabled the simultaneous assessment of protein expression, phosphorylation events and downstream signalling products, including TBK‐1 and IRF‐3 and type‐I IFN, offering a holistic view of the STING pathway's functional landscape.

## 2 Results

### 2.1 Baseline STING Pathway Expression and Signalling Dynamics Across Cell Lines

**FIGURE 1:**
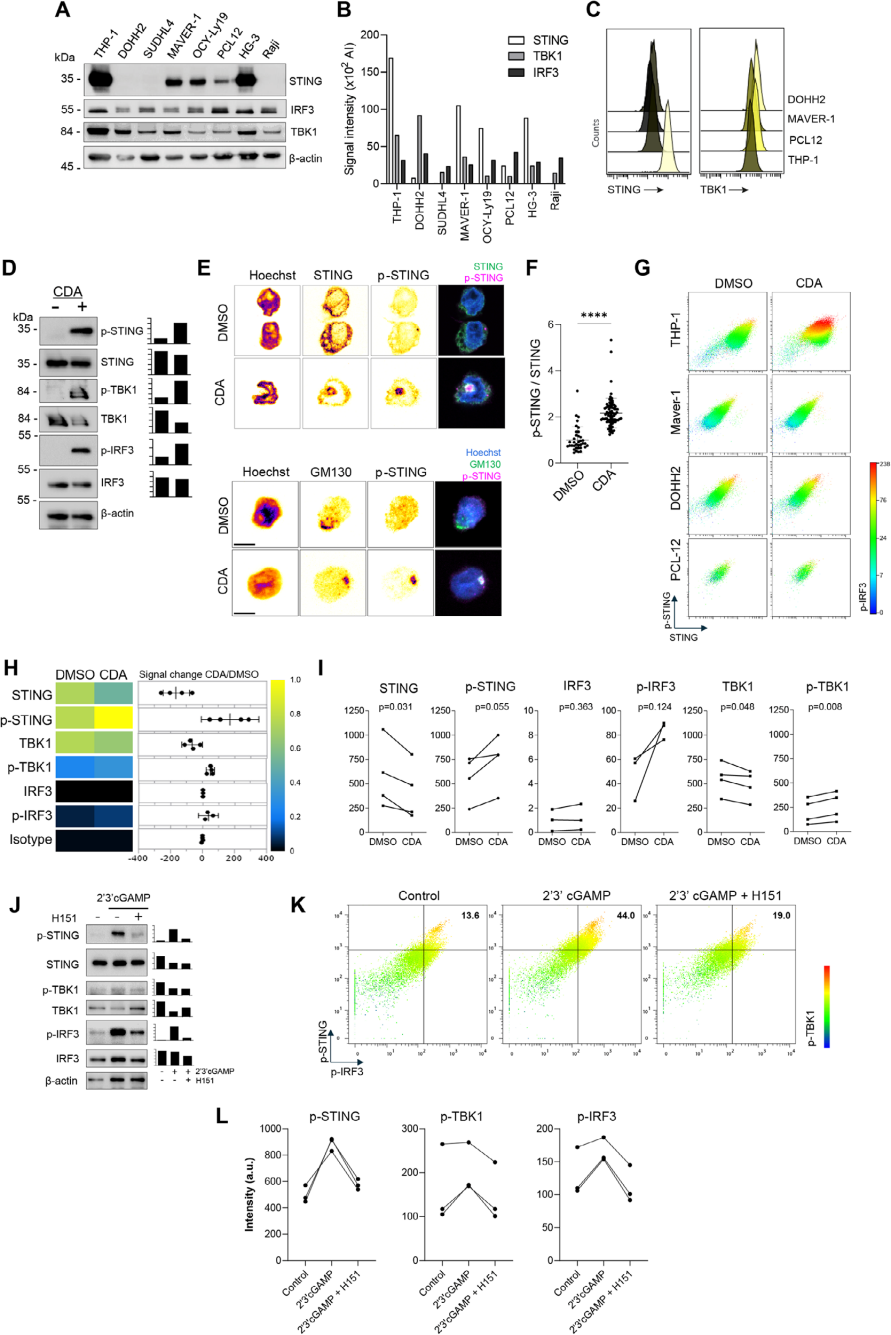
Multiparametric profiling of STING pathway components and phosphorylation states in cell lines. (A) Immunoblot analysis of STING, IRF3, TBK1 and β‐actin (loading control) in lysates from indicated cell lines prepared using RIPA buffer. (B) Densitometric quantification of protein levels normalized to β‐actin. (C) Mass cytometry measurement of STING and TBK1 expression in indicated cell lines. (D) Immunoblot analysis of STING pathway components and their phosphorylated forms in THP‐1 cells treated with 10 µg/mL CDA for 6 h. (E) Sliced confocal microscopy images of THP‐1 cells stimulated for 6 h with the indicated components then fixed and stained with the indicated markers. (F) Quantification of fluorescence intensity of phosphorylated STING (p‐STING) relative to total STING per cell, from confocal z‐stack images in 3 independent experiments; one‐way ANOVA. (G) Mass cytometry analysis of STING pathway components in THP‐1 and B cell lines treated with 10 µg/mL CDA for 6 h. p‐STING and pIRF3 are only activated in the monocytic cell line. (H) Left panel: Heatmap showing the median intensity of STING pathway components and their phosphorylated forms in THP‐1 cells treated with 10 µg/mL CDA for 6 h. Right panel and (I) Median intensities of total and phosphorylated STING pathway proteins from four independent experiments; paired *t*‐test. (J) Immunoblot analysis of STING, IRF3, TBK1 and β‐actin (loading control) in lysates from THP‐1 cells stimulated for 6 h with the STING agonist 2'3'cGAMP with or without 2 h pre‐treatment with the STING inhibitor H‐151. (K) Representative mass cytometry analysis of STING pathway components in THP‐1 cells untreated, treated with 10 µg/mL 2'3'cGAMP for 6 h and with 2 h pre‐treatment with the STING inhibitor H‐151 before activation. (L) Median intensities of total, phosphorylated STING, TBK1, IRF‐3 proteins and isotype control from three independent experiments.

To establish baseline expression levels of the STING signalling axis, we evaluated the constitutive expression of STING and its downstream effectors, TBK1 and IRF3, across a panel of hematopoietic cell lines (THP1, DOHH2, SUDHL4, MAVER‐1, OCY‐Ly19, PCL‐12, HG‐3 and Raji). Immunoblot analysis revealed heterogeneous expression of STING, with detectable protein levels observed in THP1, MAVER‐1, OCY‐Ly19, PCL‐12 and HG‐3 cells (Figure 1A,B). Notably, THP1 monocytes exhibited the highest constitutive STING expression among all tested lines. In contrast, downstream components TBK1 and IRF3 were ubiquitously expressed across all cell types, albeit with variability in baseline protein abundance (Figure 1A,B). Mass cytometry further corroborated the elevated STING levels in THP1 cells compared to other lines (Figure 1C), prompting their selection for subsequent pathway activation studies using the synthetic STING agonist, a cyclic dinucleotide analogue ML‐RR‐S2 CDA (CDA).

Stimulation of THP1 cells with CDA, triggered phosphorylation of STING (p‐STING), TBK1 and IRF3 (Figure 1D), confirming functional activation of the pathway. To delineate the spatiotemporal dynamics of STING signalling, we next employed immunofluorescence microscopy. In CDA‐treated cells, STING exhibited localisation to the Golgi apparatus, as evidenced by co‐staining with GM130, a canonical Golgi matrix protein with intensified STING phosphorylation (p‐STING) fluorescent signals concentrated at the Golgi compartment (Figure 1E,F). This spatial restriction to the Golgi underscores its role as a critical signalling hub for STING activation. Mass cytometry analysis provided high‐dimensional validation of CDA‐induced signalling events. Relative to DMSO controls, CDA‐treated THP1 cells exhibited robust p‐STING, TBK1 (p‐TBK1) and IRF3 (p‐IRF3) (Figure 1G–I), aligning with immunoblot and microscopy findings. The concordance of these techniques‐ immunofluorescence, immunoblotting and mass cytometry not only confirms the utility of mass cytometry for monitoring STING pathway activity but also reinforces the Golgi's centrality in coordinating downstream signalling.

In addition, we also stimulated THP1 cells with the endogenous ligand 2'3'‐cGAMP and observed activation of STING pathway by western blotting and mass cytometry (Figure 1J–L), confirming functional activation of the pathway, thereby validating our platform's ability to detect pathway activation by structurally diverse agonists. Furthermore, treatment with STING palmitoylation inhibitor H‐151 abrogated the p‐STING, TBK1 and IRF3 induced by 2'3'‐cGAMP (Figure 1J–L), confirming the critical dependence of this signalling cascade on STING palmitoylation and demonstrating our method's sensitivity in detecting pathway inhibition. Collectively, our results demonstrate that while core STING pathway components (TBK1, IRF3) are broadly expressed across hematopoietic lineages, STING itself shows cell type‐specific expression patterns. The pronounced baseline STING levels in THP1 monocytes, coupled with their responsiveness to diverse agonists and inhibition by H‐151, highlight the utility of this platform for probing the STING pathway and the influence of cellular context on signalling capacity.

### 2.2 Effect of STING Activation in PBMC Subpopulations

**FIGURE 2:**
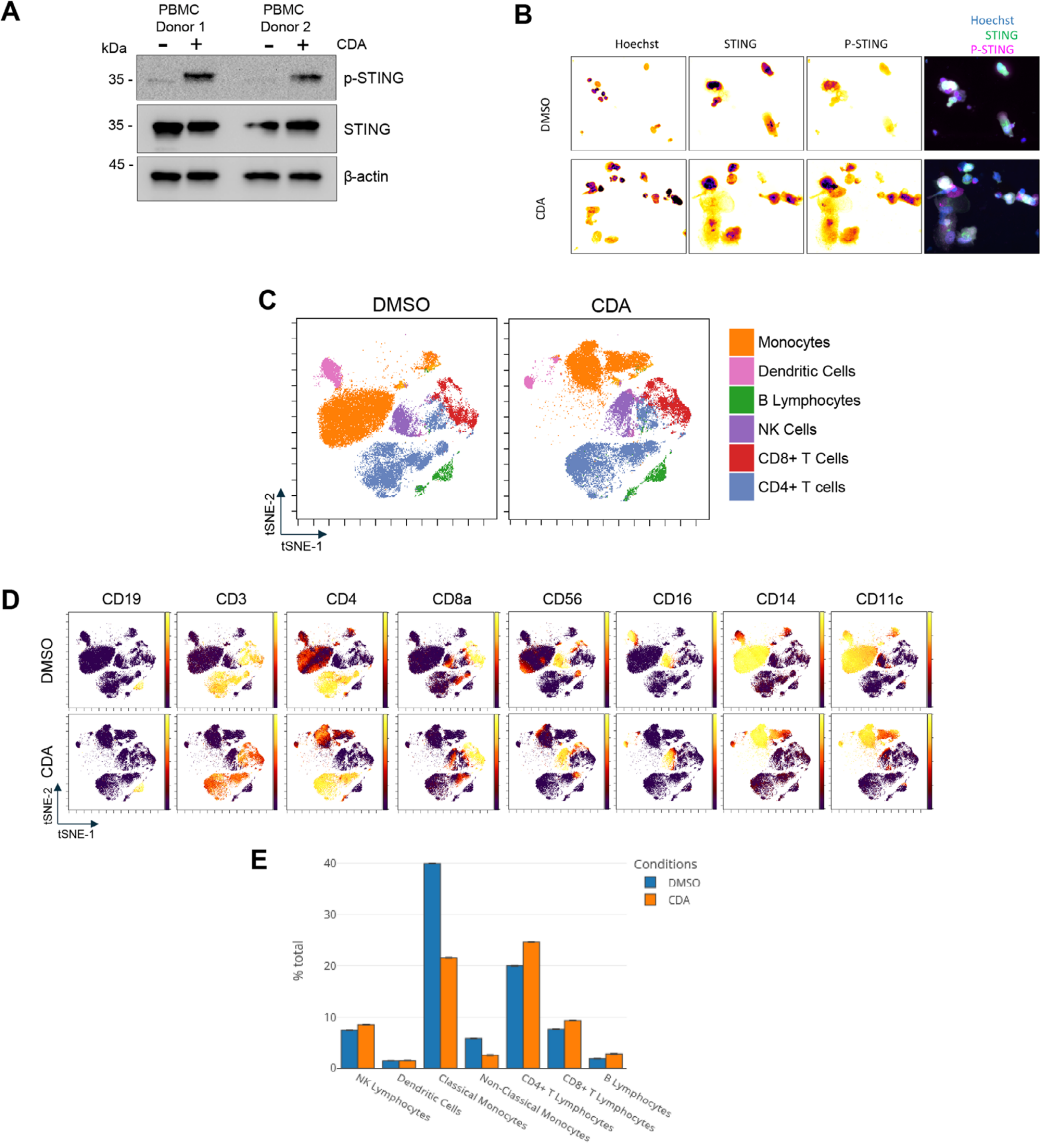
Multiparametric profiling of STING pathway activation in PBMCs. (A) Immunoblot analysis of STING, pSTING and β‐actin (loading control) in lysates from two PBMC donors. Right panel: Densitometric quantification of protein levels normalized to β‐actin. (B) Stacked confocal microscopy images of PBMCs stimulated for 6 h with the indicated components, then fixed and stained for STING, pSTING and Hoechst. (C) Overlay of FlowSOM‐identified metaclusters onto a viSNE (t‐distributed stochastic neighbour embedding) map, showing major PBMC populations following 6 h stimulation with DMSO (upper panel) or CDA (lower panel). The monocyte lineage shows a visual shift, indicating a strong effect of STING activation. (D) Mean lineage marker intensity visualized in vi‐SNE. (E) Comparison of the relative proportion of each lineage in control versus STING‐activated PBMCs.

To extend these observations to a physiologically relevant model, we evaluated CDA‐mediated STING activation in primary human peripheral blood mononuclear cells (PBMCs) from healthy donors. Immunoblot analysis (Figure 2A, *n* = 6, two donors shown) and immunofluorescence microscopy confirmed robust p‐STING in PBMCs following CDA treatment (Figure 2B). Mass cytometry profiling of PBMCs delineated major immune subsets, including monocytes (CD14⁺CD16^−^ classical and CD14^−^CD16⁺ non‐classical), dendritic cells (DCs), B cells, CD4⁺ T cells, CD8⁺ T cells and NK cells, as shown by t‐SNE visualization (Figure 2C). Strikingly, CDA treatment induced profound and selective changes in immune cell viability and frequency. Both classical and non‐classical monocytes exhibited the most dramatic response: their relative abundance decreased by ∼50% post‐stimulation (Figure 2D,E), a finding concordant with reports linking STING hyperactivation in monocytes to caspase‐dependent apoptosis [11]. In contrast, DCs and lymphocytes (B cells, T cells, NK cells) showed no significant reduction in viability, underscoring the heterogeneity of cellular responses to STING agonism. The preferential depletion of classical monocytes aligns with their high basal STING expression and unique susceptibility to STING‐driven apoptosis, a mechanism previously attributed to prolonged TBK1‐IRF3 signalling and subsequent PARP‐1 cleavage [12]. This cell type‐specific cytotoxicity suggests that STING activation may differentially regulate immune subset survival, potentially shaping inflammatory or tolerogenic responses in vivo. Notably, the resilience of lymphocytes to CDA‐induced death implies alternative regulatory checkpoints or lower STING activity thresholds in these populations.

### 2.3 Cell Type‐Specific STING Pathway Activation and Functional Responses in Primary Immune Subsets

**FIGURE 3:**
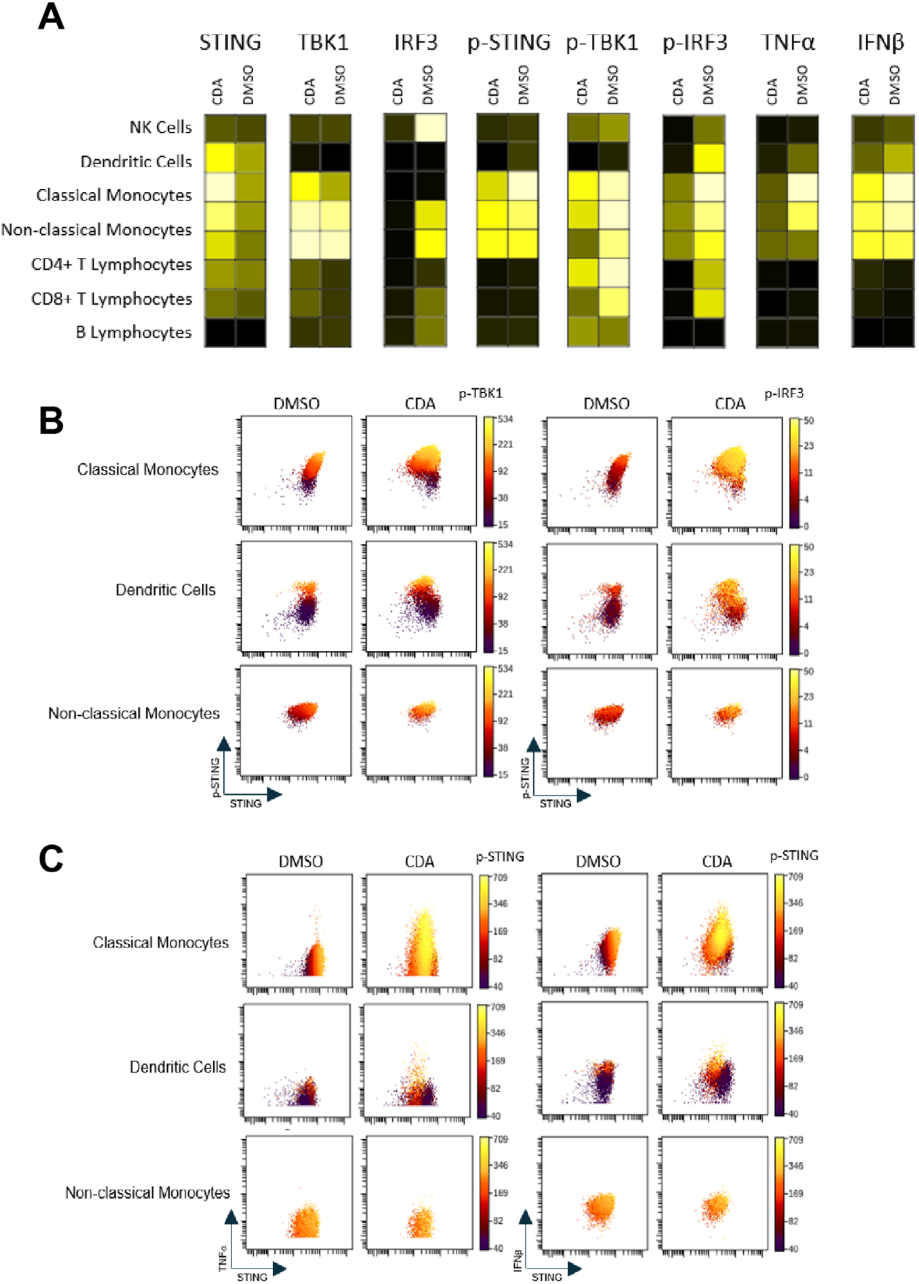
Mass Cytometry profiling of STING pathway components and phosphorylation states in PBMCs. (A) Heatmap showing differential expression of STING pathway proteins across PBMC populations, indicating that STING is expressed across all lineages except B lymphocytes. After 6 h treatment with a STING agonist, the pathway is activated in monocytes, dendritic cells (DCs), T lymphocytes and natural killer (NK) cells, as evidenced by increased median intensity of phosphorylated forms. STING activation induces Type‐I interferon expression profoundly in myeloid cells. (B) and (C) Triaxial graph showing co‐activation of STING, TBK1 and IRF3 via their phosphorylated forms in monocytes and DCs following CDA activation (B) and strong induction of TNF‐α and IFN‐β in the classical monocyte population (C). *n* = 3, representative experiment.

Mass cytometry analysis of CDA‐treated PBMCs delineated striking cell type‐specific activation patterns within the STING pathway. Monocytes emerged as the dominant responders, exhibiting robust p‐STING, TBK1 (p‐TBK1) and IRF3 (p‐IRF3), alongside elevated production of Type‐I interferon‐beta (IFN‐β) and the proinflammatory cytokine tumour necrosis factor‐alpha (TNF‐α) (Figure 3A). DCs displayed a more moderate response, with detectable p‐STING pathway components and intermediate IFN‐β and TNF‐α secretion (Figure 3A). In contrast, B cells showed no activation of STING or its downstream effectors, consistent with prior reports demonstrating negligible STING expression in healthy, uninfected B cells [13] (Figure 3A). Intriguingly, while low levels of STING protein were detected in T cell subsets (CD4⁺ and CD8⁺), no significant phosphorylation of TBK1 or IRF3 was observed, suggesting either insufficient STING expression or active suppression of signalling in these populations (Figure 3A).

Visualisation of single‐cell responses via viSNE plots reinforced these findings, revealing distinct clusters of p‐STING⁺, p‐TBK1⁺ and p‐IRF3⁺ cells predominantly localized to monocyte and DC populations (Figure 3B). Similarly, cytokine mapping demonstrated IFN‐β and TNF‐α production almost exclusively within these subsets (Figure 3C). The pronounced responsiveness of monocytes aligns with their role as frontline innate immune sensors, while the muted response in T cells, despite detectable STING hints at cell type‐specific regulatory mechanisms, such as differential expression of negative regulators or preferential signalling through alternative pathways.

## 3 Discussion

This study establishes mass cytometry as a transformative tool for dissecting the complexity of the STING pathway in heterogeneous cellular systems, overcoming critical limitations of traditional methodologies. While immunoblotting and qPCR remain foundational for studying bulk protein or transcript levels, their inability to resolve cell type‐specific signalling dynamics in mixed populations has hindered progress in understanding context‐dependent STING biology. By leveraging CyTOF's high‐dimensional, single‐cell resolution, we systematically mapped STING pathway activation across immune lineages, capturing both its baseline expression and agonist‐induced functional responses with higher granularity. Crucially, we demonstrated that our platform yields consistent and robust results across structurally distinct STING agonists, as evidenced by the phosphorylation of key proteins induced by the synthetic cyclic dinucleotide CDA and the endogenous ligand 2'3'‐cGAMP. Our findings not only validate CyTOF as a robust platform for studying innate immune signalling but also reveal its unique capacity to uncover cellular heterogeneity, pathway crosstalk and functional outcomes that conventional techniques obscure.

A key advantage of CyTOF lies in its ability to simultaneously quantify multiple signalling nodes (e.g., p‐STING, p‐TBK1, p‐IRF3) alongside phenotypic markers and functional outputs (e.g., cytokines, apoptosis) within the same experiment. This capability was further validated through mechanistic inhibition, pre‐treatment with the palmitoylation blocker H‐151 completely abrogated phosphorylation across all three nodes, functionally linking the detected phospho‐signals to this crucial upstream post‐translational modification and underscoring the platform's sensitivity to pharmacological perturbation. This multiplexed approach enabled us to correlate STING activation status with cell identity and viability in primary PBMCs, revealing that monocytes, despite their high STING expression and robust phosphorylation cascades [8, 14, 15], are selectively depleted upon CDA stimulation. In contrast, DCs, while responsive to CDA, exhibited resilience to cell death, suggesting divergent regulatory checkpoints even within myeloid subsets. Such insights would be inaccessible to immunoblotting, which homogenizes cellular responses, or flow cytometry, which lacks the metal‐isotope resolution to track > 40 parameters without spectral overlap.

Our data also underscore the importance of single‐cell analysis in resolving conflicting literature reports. For instance, while bulk assays have historically suggested negligible STING activity in lymphocytes, our CyTOF‐based approach confirmed that healthy B cells lack STING, whereas T cells retain low‐level expression without downstream signalling. This aligns with clinical observations that B cell malignancies occasionally acquire STING activity post‐infection [13], highlighting the need for techniques that differentiate baseline states from context‐dependent adaptations. Similarly, the spatial resolution afforded by immunofluorescence showing that STING activation is confined to the Golgi even in responsive cells [16, 17], complemented CyTOF's molecular granularity, reinforcing the value of integrating orthogonal methods to map signalling architecture.

The clinical implications of this work are particularly salient for STING‐targeted therapies in cancer. Current agonists often fail to account for the stark variability in STING expression and function across immune subsets, leading to unpredictable efficacy or toxicity [18]. CyTOF's capacity to profile patient‐specific immune landscapes could guide biomarker‐driven strategies, such as enriching for STING‐high monocytes or DCs in adoptive cell therapies, while avoiding populations prone to apoptosis. Furthermore, coupling CyTOF with adaptive immune markers (e.g., checkpoint proteins, exhaustion signatures) could elucidate how STING activation modulates antitumour T cell responses, a critical gap in understanding why some patients respond to STING agonists despite limited lymphocyte engagement.

In conclusion, this study highlights CyTOF as an important approach for advancing STING pathway research, providing a blueprint to dissect its roles in infection, autoimmunity and cancer. By transcending the limitations of bulk assays, CyTOF empowers researchers to unravel cellular decision‐making in vivo, from signal transduction to cytokine secretion and cell fate.

### 3.1 Data Limitations and Perspectives

This study is focused on establishing a CyTOF‐based methodology for analysing the core canonical STING‐TBK1‐IRF3 axis in human hematopoietic cells. Consequently, the presented data have inherent limitations. First, the platform's application is demonstrated in PBMCs and cell lines. We have not extended it to non‐hematopoietic tissues, as this would require extensive optimisation of species‐specific and tissue‐specific digestion protocols beyond the scope of this methodological resource. Second, our panel was specifically designed to avoid non‐specific pathways. Therefore, we excluded analysis of NF‐κB activation, as its induction is a convergent point for numerous signalling cascades and cannot be definitively attributed solely to STING activation in a complex primary cell milieu. While this may seemingly narrow the biological scope, it ensures the precision and interpretability of the single‐cell phospho‐signalling data we present. Future work will be required to adapt this platform for broader cellular and pathway contexts.

## 4 Methods

### 4.1 Cell Lines, Culture Conditions and Reagents

The human hematopoietic cell lines THP‐1 (RRID:CVCL_0006), DoHH2 (RRID:CVCL_1179), SU‐DHL‐4 (RRID:CVCL_0539), MAVER‐1 (RRID:CVCL_1831), OCI‐Ly19 (RRID:CVCL_1878), PCL12 (RRID:CVCL_2H32), HG‐3 (RRID:CVCL_Y547) and Raji (RRID:CVCL_0511) were sourced from the American Type Culture Collection (ATCC) or the German Collection of Microorganisms and Cell Cultures (DSMZ). Cell lines were cultured in RPMI 1640 Medium, GlutaMAX Supplement (Thermo Fisher Scientific, #61870044) supplemented with 10% heat‐inactivated Fetal Bovine Serum (Thermo Fisher Scientific, #A5256701), 100 U/mL penicillin and 100 µg/mL streptomycin (Thermo Fisher Scientific, #15140122). Cell viability and concentration were assessed using a Cellometer Auto T4 (Nexcelom Bioscience & Peqlab Biotechnologie GmbH) and Trypan Blue staining (Sigma‐Aldrich, #93595). Cells were passaged every 3–4 days by dilution to 0.3–0.5 million cells/mL in a humidified 5% CO_2_ atmosphere at 37°C. Mycoplasma contamination was routinely assessed using qPCR. For experiments, the STING agonist ADU‐S100 “ML RR‐S2 CDA” (MedChemExpress, #HY‐12885) was dissolved in DMSO and applied at 10 µg/mL for 6 h. The STING agonist 2′3′‐cGAMP (Invivogen, #tlrl‐nacga23‐02) was dissolved in water and applied at 10 µg/mL for 6 h. The STING antagonist H‐151 (Tocris Bio‐Techne, #6675) was dissolved in DMSO and applied at 0.5 µg/mL for 2 h prior to experiments.

### 4.2 PBMC Isolation

PBMCs were isolated from leukocyte cones (NHS Blood and Transplant, UK) using Lymphoprep (StemCell Technologies, #07851) density gradient centrifugation according to manufacturer instructions. Briefly, blood diluted 1:4 with PBS containing 10% FBS was gently layered over 12.5 mL of room temperature Lymphoprep in 50 mL tubes. Samples were centrifuged at 800 × g for 20 min at RT with low brake. The PBMC layer was carefully harvested using a Pasteur pipette and washed twice with PBS. Cells were counted using a Cellometer Auto T4 and trypan blue exclusion. PBMCs were resuspended in FBS containing 10% DMSO (Fisher Chemical, #D/4120/PB08) at 50–100 million cells/mL, aliquoted into cryovials and kept in liquid nitrogen for long‐term storage. Before use, PBMCs were thawed at 37°C, diluted in pre‐warmed culture medium and washed before viability assessment.

### 4.3 Immunoblotting

Cells were lysed in Radio‐Immunoprecipitation Assay (RIPA) buffer (Sigma‐Aldrich, Merck KGaA, Darmstadt, Germany, #R0278) supplemented with Halt Protease and Phosphatase Inhibitor Cocktail (100X, Thermo Fisher Scientific, #78440), kept on ice, sonicated for 60 s (Sonopuls, Bandelin) and centrifuged at 12,000 × g for 5 min. Protein concentrations were determined from the supernatant using the Pierce BCA Protein Assay Kit (Thermo Fisher Scientific, #23227), measuring absorbance at 562 nm (μQuant, Biotek). Lysates were stored at ‐80°C until further use.

Prior to electrophoresis, lysates were thawed, diluted in Western‐Ready Protein Sample Loading Buffer (5X, BioLegend, #426311) or 4X Laemmli Sample Buffer (Bio‐Rad, #1610747), and incubated at 95°C for 5–10 min to denature proteins. Equal amounts of protein were loaded onto 10% SDS‐PAGE gels and transferred to nitrocellulose membranes. Membranes were blocked with 5% BSA or non‐fat milk in TBS‐T and incubated overnight at 4°C with primary antibodies against phospho‐STING, total STING, phospho‐TBK1, total TBK1, phospho‐IRF3, total IRF3 and actin. After washing, membranes were incubated with HRP‐conjugated secondary antibodies, and chemiluminescent signals were detected using ECL‐Advance Western Blotting Detection reagents (Millipore, Fisher Scientific UK Ltd, #WBKLS0500) on a ChemiDoc MP imaging system (Bio‐Rad). Band intensities were quantified using Fiji (NIH, Schindelin et al.) following background subtraction and were normalised to loading controls.

### 4.4 Mass Cytometry (CyTOF)

#### 4.4.1 Antibodies

Antibodies were either acquired pre‐conjugated or labelled in‐house using Maxpar X8 Multimetal Labeling Kits or MCP9 polymer kits (Standard BioTools), according to the manufacturer's guidelines. The panel included the following key phospho‐specific antibodies to monitor STING pathway activation: anti‐phospho‐STING (S366), anti‐phospho‐TBK1 (S172) and anti‐phospho‐IRF3 (S396). Cell line and PBMC samples from different activation conditions were incubated for 30 min at 4°C with CD45 antibodies conjugated to different combinations of 106Cd, 110Cd, 111Cd, 113Cd, 114Cd, 115In and 116Cd. Barcoded samples were then washed twice (500 × g, 4°C, 5 min) with Maxpar Cell Staining Buffer (CSB; Standard BioTools) and pooled into a single multiplexed sample.

#### 4.4.2 Staining

Pooled samples were incubated for 5 min at room temperature with Cell‐ID Cisplatin (Fluidigm, #201064) for viability staining, then washed in CSB. Surface staining was performed by incubating the cells for 45 min at 4°C with a cocktail of diluted antibodies targeting surface epitopes. After washing, cells were fixed in 1.6% formaldehyde (Thermo Fisher Scientific, #28906) for 15 min at 4°C and washed twice in CSB. Intracellular staining was performed by incubating cells for 60 min at 4°C with an antibody mix for intracellular epitopes. Following washes in CSB, cells were incubated with Cell‐ID Intercalator‐Ir (Standard BioTools, #201192A), diluted 1:2000 in 1.6% formaldehyde and stored at 4°C for up to 48 h.

#### 4.4.3 Acquisition

Cells were washed with Maxpar Cell Acquisition Solution (Standard BioTools, #201237), then resuspended with EQ Four Element Calibration Beads (Standard BioTools, #201078) at a 1:10 ratio. Samples were acquired on a Helios Mass Cytometer (Standard BioTools), with events recorded at a rate of 200–400 cells/s. Data were normalized using the CyTOF Software v8.0 acquisition software and exported as .fcs files.

### 4.5 CyTOF Data Analysis

Normalized raw data were analysed using the online Cytobank platform (Beckman Coulter Life Sciences, US). The gating strategy to identify live singlets was as follows: debris and doublets were excluded by gating on Intercalator‐Ir and Gaussian parameters, and viable cells were identified by gating out 195Pt‐positive events. Samples were manually debarcoded according to CD45 barcode staining. We used the unsupervized viSNE dimensionality reduction algorithm in Cytobank to generate two‐dimensional maps of the multiparametric CyTOF data. Because 6 h STING‐agonist treatment induces high mortality in monocytes, proportional sampling was applied. For viSNE analysis, both phenotyping and functional/activation marker channels were included to visualize the effects of STING activation. Following dimensionality reduction, clustering was performed using the FlowSOM self‐organizing map algorithm. Manual gating was also performed to identify all cell populations, including classical and non‐classical monocytes. The heatmap in Figure 1H was generated using GraphPad Prism (version 10.5.0, GraphPad Software), and the heatmap in Figure 3A was generated using Cytobank.

### 4.6 Confocal Microscopy

Indicated cell lines or PBMCs were seeded for 2 h onto coverslips pre‐coated with Poly‐L‐Lysine (0.1 mg/mL, Sigma, # L8662) and stimulated for 6 h at 37°C in 5% CO_2_ with 10 µg/mL of the STING agonist “CDA” (MedChemExpress, #HY‐12885) or DMSO (Fisher Chemical, #D/4120/PB08) as a control. Cells were fixed with 4% formaldehyde (Thermo Fisher Scientific, #28906) for 15 min at 4°C and washed three times in PBS. Cells were then blocked in 1 × PBS containing 0.1% Triton X‐100 (Fisher Chemical, #11488696) and 5% BSA for 1 h. Primary antibodies were applied overnight at 4°C, followed by secondary antibody incubation for 1 h after extensive washing. Nuclei were stained with Hoechst 33342 (Thermo Fisher Scientific, #62249) for 5 min, and coverslips were mounted using ProLong Gold Antifade Reagent (Invitrogen, Thermo Fisher Scientific, #P36930). Confocal z‐stack images were acquired using a Zeiss LSM 800 Airyscan or LSM 900 Airyscan2 microscope and processed with ZEN Blue software (Zeiss). Fluorescence intensities of stacked confocal images were quantified per cell using Fiji (NIH, Schindelin et al.) after background subtraction within the same field of view.

## Author Contributions

AR, PTK and AG conducted experiments, acquired, analysed and interpreted data. JRS provided material support, reagents and intellectual input. LND designed the study, analysed and interpreted data and wrote the manuscript. All authors read, edited and approved the final manuscript.

## Conflicts of Interest

The authors declare no conflicts of interest.

## Data Availability

The data that support the findings of this study are available from the corresponding author upon reasonable request.
